# Respiratory syncytial virus infection changes the piwi-interacting RNA content of airway epithelial cells

**DOI:** 10.3389/fmolb.2022.931354

**Published:** 2022-09-08

**Authors:** Tiziana Corsello, Andrzej S Kudlicki, Tianshuang Liu, Antonella Casola

**Affiliations:** ^1^ Department of Pediatrics, The University of Texas Medical Branch at Galveston (UTMB), Galveston, TX, United States; ^2^ Institute for Translational Sciences, The University of Texas Medical Branch at Galveston (UTMB), Galveston, TX, United States; ^3^ Department of Biochemistry and Molecular Biology, The University of Texas Medical Branch at Galveston (UTMB), Galveston, TX, United States; ^4^ Department of Microbiology and Immunology, The University of Texas Medical Branch at Galveston (UTMB), Galveston, TX, United States

**Keywords:** piwi-interacting RNA, viral infection, airways, RSV, epithelial cells

## Abstract

Piwi-interacting RNAs (piRNAs) are small non-coding RNAs (sncRNAs) of about 26–32 nucleotides in length and represent the largest class of sncRNA molecules expressed in animal cells. piRNAs have been shown to play a crucial role to safeguard the genome, maintaining genome complexity and integrity, as they suppress the insertional mutations caused by transposable elements. However, there is growing evidence for the role of piRNAs in controlling gene expression in somatic cells as well. Little is known about changes in piRNA expression and possible function occurring in response to viral infections. In this study, we investigated the piRNA expression profile, using a human piRNA microarray, in human small airway epithelial (SAE) cells infected with respiratory syncytial virus (RSV), a leading cause of acute respiratory tract infections in children. We found a time-dependent increase in piRNAs differentially expressed in RSV-infected SAE cells. We validated the top piRNAs upregulated and downregulated at 24 h post-infection by RT-qPCR and identified potential targets. We then used Gene Ontology (GO) tool to predict the biological processes of the predicted targets of the most represented piRNAs in infected cells over the time course of RSV infection. We found that the most significant groups of targets of regulated piRNAs are related to cytoskeletal or Golgi organization and nucleic acid/nucleotide binding at 15 and 24 h p.i. To identify common patterns of time-dependent responses to infection, we clustered the significantly regulated expression profiles. Each of the clusters of temporal profiles have a distinct set of potential targets of the piRNAs in the cluster Understanding changes in piRNA expression in RSV-infected airway epithelial cells will increase our knowledge of the piRNA role in viral infection and might identify novel therapeutic targets for viral lung-mediated diseases.

## Introduction

Respiratory syncytial virus (RSV) is a single-stranded RNA virus that belongs to the family of Pneumoviridae (Rima et al., 2017) and is the leading cause of acute lower respiratory tract infections in children worldwide, and it also results in severe respiratory infections in the elderly and immunocompromised patients (Shi et al., 2017; Shi et al., 2020). An effective treatment or vaccine is not currently available for RSV infection, and many molecular mechanisms regarding RSV-induced lung disease are still unknown (Mazur et al., 2015; Domachowske et al., 2021). Piwi-interacting RNAs (piRNAs) are a novel class of small non-coding RNAs (sncRNAs) which are between 26 and 32 nucleotides in length and have a 2′-O-methylated residue on their 3′ terminal base (Kirino and Mourelatos, 2007 (Fu and Wang, 2014). piRNAs can be grouped into four classes according to their origin: 1) transposon—derived piRNAs, coming from RNA transcripts of active transposable element copies; 2) piRNAs originated from the three untranslated regions (UTRs) of messenger RNAs (mRNAs); 3) non-coding RNAs derived piRNAs and 4) Caenorhabditis piRNAs found in worms with unique properties (Han and Zamore, 2014; Yang et al., 2016; Ernst et al., 2017), although their biogenesis is still not fully understood. piRNAs interact with the P-element Induced Wimpy Testis (PIWI) proteins, forming RNA-protein complexes known as piwi-interacting RNA complex (piRC). The piRC are involved in multiple cellular functions as gene regulation, chromatin modifications and transposon silencing in different organisms (Iwasaki et al., 2016; Monga and Banerjee, 2019). piRNAs were originally discovered in germ cells, maintaining the genome integrity and repressing mutations via transposable elements (Weick and Miska, 2014). Recently, they have been characterized in multiple organs and somatic cells (Zamore, 2010; Théron et al., 2014), and their unique expression profile is associated with the development of several diseases, including cancer, diabetes, cardiovascular and neurodegenerative disorders (Moyano and Stefani, 2015; Wakisaka and Imai, 2019; Wu et al., 2020; Rayford et al., 2021; Zeng et al., 2021). However, little is known about changes in piRNA expression and possible function occurring in response to viral infections. In our previous published work, we identified piRNAs among the sncRNAs present in extracellular vesicles altered by infection of airway epithelial cells with RSV (Chahar et al., 2018). In this study, we examined the changes in piRNA profile in primary small airway epithelial (SAE) cells infected with RSV, using a human piRNA microarray. We found that RSV infection of SAE cells was associated with a time-dependent increase in differentially expressed piRNAs, when compared to uninfected cells. Among the detected piRNAs, we validated six of the top ten upregulated at 24 h p. i. By RT-qPCR method. We used Gene Ontology (GO) analysis to predict the biological processes, molecular function and cell components of the most represented predicted targets of piRNAs over the three time points and we performed cluster analysis of the expression profiles. Identification of human piRNAs and their targets in airway epithelial cells will help to uncover new roles of this class of sncRNAs following viral infections, and to identify possible biomarkers and/or novel therapeutic targets for viral-mediated lung diseases.

## Materials and methods

### Small airway epithelial (SAE) culture and Respiratory syncytial virus infection

SAE cells (Lonza Inc., San Diego, CA), derived from terminal bronchioli of two different cadaveric donors (25- and 38-years old donors), were grown in growth medium, containing 7.5 mg/ml bovine pituitary extract (BPE), 0.5 mg/ml hydrocortisone, 0.5 μg/ml hEGF, 0.5 mg/ml epinephrine, 10 mg/ml transferrin, 5 mg/ml insulin, 0.1 μg/ml retinoic acid, 0.5 μg/ml triiodothyronine, 50 mg/ml gentamicin and 50 mg/ml bovine serum albumin. For preparation of RSV stocks, RSV Long strain was grown in Hep-2 cells and purified by centrifugation on discontinuous sucrose gradient as described previously (Ueba, 1978). SAE cells were switched to basal media (not supplements added) prior to RSV infection. At 90%–95% confluence, cell monolayers were infected with RSV at multiplicity of infection (MOI) of 3. An equivalent amount of 30% sucrose solution was added to uninfected SAE cells as a control (mock cells). Cells were collected at 6, 15 and 24 h RSV post-infection (p.i.) for the next analysis. We used two different donors and three replicates per time point.

### Total RNA extraction and Piwi-interacting RNAs microarray

Total RNA was extracted using ToTALLY RNA kit from Ambion (Life Technologies). piRNA microarray analysis was performed by Go Beyond RNA Company (Arraystar Inc., Rockville, MD). RNA samples were quantified using a Nanodrop Spectrophotometer (Nanodrop Technologies) and integrity was assessed using standard denaturing agarose gel electrophoresis. The Arraystar HG19 piRNA array was designed for profiling of piRNAs in human cells. Human piRNAs were downloaded from the NCBI database and mapped to the HG19 genome sequence using UCSC Blat. DQ (accession ID) was used to search exact piRNA in the GenBank sequences, NCBI database (Wang et al., 2019). piRNAs with good match were selected, and on this array, probes of about 23,000 piRNAs were designed successfully using a duplex method. piRNA sample labeling was performed using an RNA ligase method. Briefly, 1 μg of each sample was 3′-end-labeled with Cy3 fluorescent label, using T4 RNA ligase using the following procedure: RNA in 2.0 μl of water was combined with 1.0 μl of CIP buffer and CIP (Exiqon). The mixture was incubated for 30 min (min) at 37°C, and was terminated by incubation for 5 min at 95°C. Then 3.0 μl of labeling buffer, 1.5 μl of fluorescent label (Cy3), 2.0 μl of DMSO, 2.0 μl of labeling enzyme were added into the mixture. The labeling reaction was incubated for 1 h at 16°C, and terminated by incubation for 15 min at 65°C. After stopping the labeling procedure, the Cy3-labeled samples were hybridized on the Arraystar Human piRNA Array. Hybridization was performed at 65°C for 18 h in Agilent’s SureHyb Hybridization Chambers. After being washed in an ozone-free environment, the slides were fixed and scanned using the Agilent DNA Microarray Scanner.

### Data analysis of the Piwi-interacting RNAs microarray

Agilent Feature Extraction software (version 11.0.1.1) was used to analyze the acquired array images. Quantile normalization and subsequent data processing were performed using the GeneSpring GX v12.1 software package (Agilent Technologies). After quantile normalization of the raw data, piRNAs with Present or Marginal calls (microarray quality control flags) for at least four out of the 24 samples (six replicates for four time points) were chosen for further data analysis. Consistency between the two groups of three replicates at each time point was confirmed by visual inspection of the corresponding scatterplot. Differentially expressed piRNAs were identified through Fold Change filtering, combined with paired *t*-test across the six replicates. The *t*-test analysis was performed on log-transformed expression values. The use of logarithmic transformation is justified based on the skewness test (mean skewness of log transformed data k_3_ = −0.3, which signifies only moderate deviation from normal distribution). Differentially expressed piRNAs between two groups were defined through a cut-off fold ratio, and significance, equivalent to Volcano Plot filtering. The resulting data were listed as fold ratios and *p*-values.

### Predicted targets of Piwi-interacting RNAs and functional analysis

Prediction of potential targets of the piRNAs was performed by combining piRNA alignments with lists of genomic features, using the bedtools package. To annotate the potential targets of the identified piRNAs, we used the genomic coordinates of the piRNAs, as available from the piRNAQuest resource, http://bicresources.jcbose.ac.in/zhumur/pirnaquest/, (Ghosh et al., 2022). We used the UCSC liftOver tool (Hinrichs et al., 2006) and BEDTools (Quinlan and Hall, 2010) to identify overlaps of piRNA sequences with genomic features, as in the piRNAdb database. We verified the implementation by comparing selected results against piRNAdb. We used Panther version 17.0 to analyze the Gene Ontology (GO) annotations and enrichments within the significant predicted target genes for the differentially active piRNAs between each two timepoints (*p* value <0.01 for differential expression). The cutoff for calling enrichments in Panther was set as FDR <0.05 for Fisher exact test. We examined the three primary GO categories: biological process, molecular function, and cell component. Relative frequencies were calculated against the entire human genome as background.

### Reverse transcription quantitative PCR (RT-qPCR)

Briefly, 600 ng total RNA containing the piRNA was reversed transcribed into cDNA in 1X RT buffer, 50 nM RT primers (Table 1), 250 nM dNTPs, 0.6 U RNase Inhibitor, and 3 U M-MuLV Reverse Transcriptase in a 20 μl reaction volume. The reaction was incubated at 16°C for 30°min, 42°C for 40°min, and 85°C for 5 min 20°μl of the synthesized cDNA was diluted in 100 μL water. The 2 μl cDNA dilution was mixed with 5 μl Arraystar SYBR^®^ Green Real-time qPCR Master Mix and 0.5 μl of 10 μM Forward and Reverse PCR primers (Table 2) in a 10 μL reaction volume. The qPCR was carried out in QuantStudio5 Real-time PCR System (Applied Biosystems) by one cycle of (95°C for 10 min) and 40 cycles of (95°C for 10°s, 60°C for 1°min, optical reading). U6 RNA was used as the housekeeping gene reference. ΔΔCt method was used to calculate the differential expression. Reverse transcription was performed using the First Strand cDNA Synthesis Kit (Arraystar). Quantitative PCR was conducted using the SYBR Green qPCR Master Mix (Arraystar), according to the manufacturer’s instructions. Primer sequences are available upon request. For the viral replication quantification, RSV N gene was amplified using specific primers, as previously described (Li et al., 2015).

**TABLE 1:** Top 10 highly up- (≥2 fold) and down-regulated (≤2 fold) piRNAs by RSV 6 h p.i (*p* ≤ 0.01). DQ is the accession number of piRNA in the NCBI database.

Name	Accession number	Fold change (Log2)
piR-61505	DQ595393	2.29
piR-38587	DQ600521	1.61
piR-47139	DQ579027	1.51
piR-36425	DQ598359	1.48
piR-30033	DQ569921	1.42
piR-30123	DQ570011	1.38
piR-55301	DQ588189	1.28
piR-44781	DQ576669	1.25
piR-36222	DQ598156	1.24
piR-43294	DQ575182	1.22
piR-45856	DQ577744	−2.74
piR-45855	DQ577743	−2.45
piR-45854	DQ577742	−2.29
piR-45512	DQ577400	−2.19
piR-32882	DQ582770	−2.13
piR-56993	DQ589881	−2.11
piR-31311	DQ571199	−2.08
piR-30049	DQ569937	−2.01
piR-47273	DQ579161	−2.00
piR-32318	DQ582206	−1.99

**TABLE 2:** Top 10 highly up- (≥4 fold) and down-regulated (≤4 fold) piRNAs by RSV 15 h p.i. (*p* ≤ 0.01). DQ is the accession number of piRNA in the NCBI database.

Name	Accession number	Fold change (Log2)
piR-38587	DQ600521	8.26
piR-61505	DQ595393	7.95
piR-47139	DQ579027	7.06
piR-61160	DQ595048	6.61
piR-37886	DQ599820	5.86
piR-32372	DQ582260	5.76
piR-45936	DQ583610	5.51
piR-50722	DQ577824	5.37
piR-32201	DQ582089	5.32
piR-54651	DQ587539	4.98
piR-38945	DQ600879	−2.26
piR-56993	DQ589881	−2.17
piR-32318	DQ582206	−2.12
piR-32882	DQ582770	−2.10
piR-45856	DQ577744	−2.06
piR-37981	DQ599915	−2.03
piR-45512	DQ577400	−2.00
piR-45855	DQ577743	−1.96
piR-30049	DQ569937	−1.84
piR-46025	DQ577913	−1.77

### Statistical analysis

The statistical analysis of the piRNA microarray was run using the PDL:Stats and Statistics:PointEstimation modules from CPAN. Differentially expressed piRNAs were identified for every pair of timepoints (6 h versus mock, 15 h versus mock, 24 h versus mock). Fold change of the differentially expressed piRNA by microarray were calculated as linear average or Log2 average ratio. Fold change of RT-qPCR experiments was calculated by 2^-ΔΔCt^ method and represent mean ± SEM using GraphPad Prism v4 (GraphPad 160 Software). *p* value <0.05 or 0.01 was considered statistically significant.

## Results

### RSV infection induces changes in piRNAs profile expression

**FIGURE 1:**
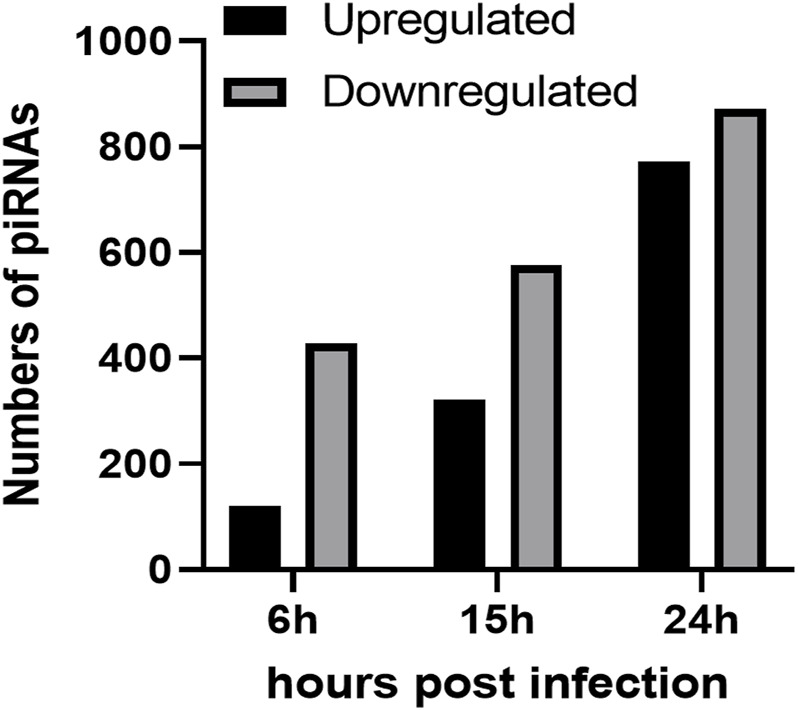
Numbers of upregulated (black bars) and downregulated (grey bars) piRNAs in RSV infected SAE cells at 6, 15 and 24 h p. i. Compared to the mock (uninfected) SAE cells. Data is representative of six replicates for each time point with *p* < 0.01.

**FIGURE 2:**
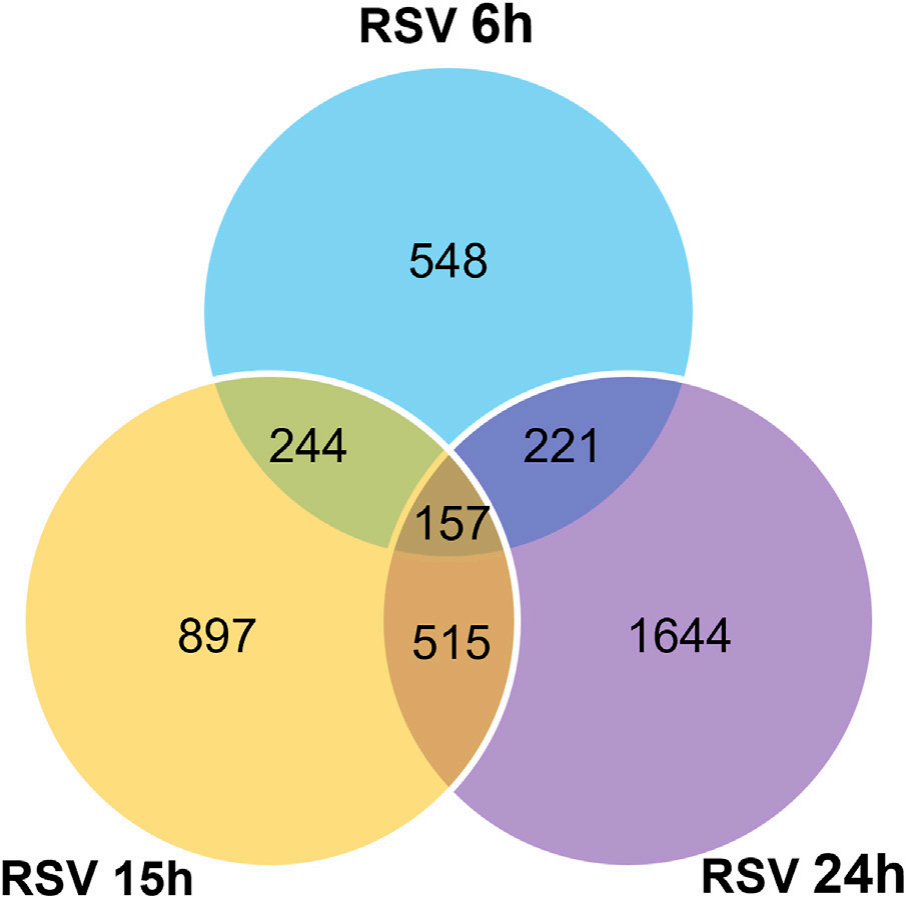
Intersecting Venn diagram showing the numbers of up- and down-regulated piRNAs differentially expressed at the three different time points (6, 15 and 24 h) RSV p. i. Compared to the mock (uninfected) SAE cells. The numbers in overlapping area(s) represent the differential regulated number of piRNAs shared among all the time points. Data is representative of six replicates for each time point with *p* < 0.01.

To investigate the impact of infection on the piRNA expression profile in primary airway epithelial cells, we analyzed piRNAs changes in human SAE cells infected with RSV for 6, 15 and 24 h p. i., using the Arraystar HG19 piRNA array, which probes for 23,677 different piRNAs. Total RNA from each sample was extracted and assessed for quality control. We measured viral repliation by detecting RSV N gene as shown in Supplementary Figure 1. Samples were labeled and hybridized to perform the piRNA microarray according to the company’s protocol. After quantile normalization and subsequent data processing, we detected 548 (6 h p.i.), 897 (15 h p.i.) and 1,644 (24 h p.i.) differentially expressed piRNAs in RSV-infected cells compared to mock (uninfected) cells. A total of 120, 321 and 772 piRNAs were significantly upregulated at 6, 15 and 24 h p. i., respectively. Interestingly, there was a higher number of downregulated piRNAs in response to viral infection, with 428, 576 and 872 differentially expressed piRNAs at 6, 15 and 24 h p. i., respectively (Figure 1). Among the differentially expressed piRNAs, 244 were shared between the time points of 6 and 15 h p.i.; 221 between 6 and 24 h p.i. And 515 between 15 and 24 h p.i., with 157 piRNAs commonly detected over all time points, as represented in the intersecting Venn diagram (Figure 2). The top ten piRNAs upregulated and downregulated (≤2 Fold) for each time point p. i. Are shown in Table 1 (6 h), Table 2 (15 h) and Table 3 (24 h). The complete list of piRNAs whose expression in SAE cells was changed in a time-dependent manner after RSV infection is shown in Supplementary files 1, two and 3.

**TABLE 3:** Top 10 highly up- (≥4 fold) and down-regulated (≤4 fold) piRNAs by RSV 24 h p.i. (*p* ≤ 0.01). DQ is the accession number of piRNA in the NCBI database.

Name	Accession number	Fold change (Log2)
piR-38587	DQ600521	9.97
piR-61505	DQ595393	9.41
piR-37886	DQ599820	8.67
piR-47139	DQ579027	8.57
piR-61160	DQ595048	8.24
piR-32372	DQ582260	8.03
piR-50722	DQ583610	7.33
piR-54651	DQ587539	7.26
piR-32201	DQ582089	7.08
piR-45936	DQ577824	6.92
piR-37981	DQ599915	−3.42
piR-42627	DQ574515	−2.85
piR-46025	DQ577913	−2.73
piR-30049	DQ569937	−2.72
piR-41627	DQ573515	−2.66
piR-38945	DQ600879	−2.64
piR-32318	DQ582206	−2.64
piR-53794	DQ586682	−2.51
piR-50660	DQ583548	−2.45
piR-60132	DQ594020	−2.31

**FIGURE 3:**
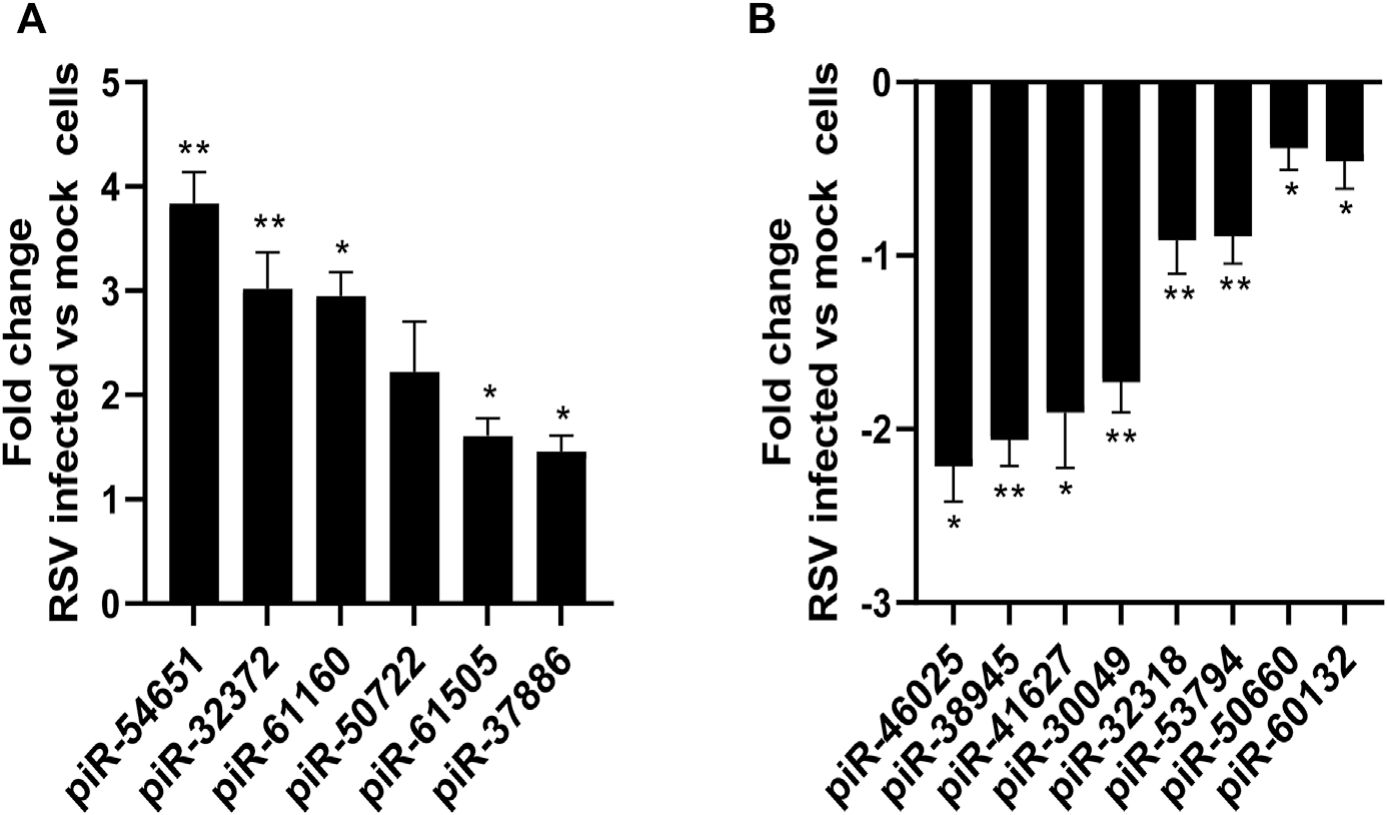
Validation of up **(A)** or down **(B)** regulated piRNAs expression in SAE cells infected with RSV at 24 h p. i. RNA extracted from SAE mock (uninfected) and RSV infected cells (24 h) was subjected to piRNAs analysis by RT-qPCR. Fold changes in piRNA expression were determined by 2^−ΔΔCT^ method and represent mean ± SEM normalized to a small nuclear RNA, U6. * and ** indicates a statistical difference comparing RSV infected versus mock cells (*p value <0.05; **p value <0.01). Data is representative of three independent experiments.

We then validated the expression of six or eight of the top ten upregulated or downregulated piRNAs at 24 h p.i. Listed in Table 3. Total RNA was isolated from SAE mock or RSV infected cells at 24 h p.i. And subjected to RT-qPCR. We found that piR-54651 (DQ587539), piR-32372 (DQ582260) and piR-61160 (DQ595048) levels increased more than threefold, and the other three piRNAs, piR-61505 (DQ595393), piR-37886 (DQ599820) and piR-50722 (DQ583610) increased one and half fold in viral-infected cells compared to mock (uninfected) cells, as shown in Figure 3A. We observed more than two fold decreased levels of piR-46025 (DQ577913) and piR-38945 (DQ600879), and the other two piRNAs, piR-41627 (DQ573515) and piR-30049 (DQ5699370) showed more than one and half fold of decreased levels in viral-infected cells compared to mock (uninfected) cells Figure 3B. In general, fold change values of piRNAs analyzed by microarray were at least two times higher than the fold change of the same piRNAs validated by RT-qPCR.

### Potential targets and Gene Ontology prediction of the viral-induced up- and down-regulated piRNAs

To characterize potential targets of piRNAs identified in RSV-infected SAE cells for each time point, we combined the piRNA alignments with lists of genomic features, using the bedtools package, and verified the approach by comparing selected results against the piRNAdb database (Piuco and Galante, 2021). At the stricter threshold for piRNA regulation at *p* < 0.01, we identified a total of 629, 1,044 and 2,750 targets, including 519, 874, and 2,118 targets of down-regulated piRNAs, and 153, 245 and 886 targets of up-regulated piRNAs, all respectively at 6, 15 and 24 h p. i. With RSV. Using the inclusive approach, we found a total of 2,874, 2,341, and 6,967 potential targets of regulated piRNAs at 6, 15 and 24 h p. i. Compared to mock (uninfected cells) with a *p* value <0.05, respectively. Of these, 1961, 1978, and 5,778 are targets of down-regulated piRNAs, and 1,313, 568, and 2,684 are targets of up-regulated piRNAs at 6, 15 and 24 h p. i. We found that most of the piRNAs had multiple target genes and that several piRNAs shared common gene targets. Potential targets of selected upregulated piRNAs following RSV infection are listed in Table 4. Most of the predicted targets belong to the class of long noncoding RNAs (lncRNAs), including long intergenic non-coding RNAs (lincRNAs) such as LINC01297, LINC01087 and LINC01087. LincRNAs are autonomously transcribed RNAs of more than 200 nucleotides in length that do not overlap protein-coding genes (Ransohoff et al., 2018). Other identified targets of the upregulated piRNAs were the endosome protein Pleckstrin Homology Domain Containing B2 (PLEKHB2), and the cytoplasm proteins the Adaptor Related Protein Complex five Subunit Zeta 1 (AP5Z1), the Probable ATP-dependent RNA helicase (DDX4), and the Signal transducer and activator of transcription 2 (STAT2). PLEKHB2 is involved in retrograde transport of recycling endosomes. AP5Z1 belongs to the family of Clathrin adaptor proteins, known also as adaptin proteins (APs), and participates in the processes of homologous recombination DNA double-strand break repair (HR-DSBR). DDX4, highly expressed in the ovary and testis, has been involved in the production of piRNAs in fetal male germ cells through a ping-pong amplification cycle, and in the secondary piRNAs metabolic process (Li et al., 2010). STAT2 protein is critical to the biological response of type I interferons (IFNs) with a DNA-binding transcription factor activity (Park et al., 2000). The complete lists of the potential target genes for each time point of RSV infection are presented in Supplementary files 1 (6 h p.i.), 2 (15 h p.i.) and 3 (24 h p.i.).

**TABLE 4:** Predicted target genes of selected upregulated piRNAs during the course of RSV infection.

piRNAs	Target gene
piR-38587	FOXK1, AP5Z1
piR-54651	PLEKHB2
piR-32372	LINC01087
piR-61160 and piR-50722	ANKRD18A and RP11-146D12.2
piR-50722	FAR2P1, LINC01297
piR-49367	SUCLG1
piR-36763	RP11-20I23.6, PDPK1, AC141586.5, PDPK2P
piR-39104	CTD-2210P24.1
piR-42026	AC093627.9
piR-53025	CAND2
piR-48109	MRPL45P1
piR-54343	RP11-753D20.1
piR-57616	NBPF11 NBPF12
piR-55323	RNF219-AS1
piR-33637	PDXDC1
piR-54439	RP11-386M24.3
piR-51361	TBC1D3P4, USP6 TBC1D3P3
piR-54546	RP1-63G5.5
piR-59040	LINC00837
piR-36425	RP11-17M15.2
piR-30123	TXNRD1
piR-55301	RP11-403P17.5, TK2
piR-44781	RP11-386M24.9
piR-36222	TRIM14
piR-43294	SQSTM1, C5orf45
piR-48124	LRIG2, RLIMP2
piR-51401	NOD2
piR-40148	TRAP1
piR-57640	STAT2
piR-49095	DDX4

To characterize the prevalent functions of the predicted targets of the differentially expressed piRNAs we use the Gene Ontology (GO) functional classification (biological process, cellular component, and molecular function). We found that the most significant groups of targets of regulated piRNAs are related to cytoskeletal or Golgi organization and nucleic acid/nucleotide binding at 15 and 24 h p.i. (*p* < 0.01). A summary of the GO biological process of the predict targets of piRNAs upregulated at 24 h RSV p.i (*p* < 0.05) is listed in Table 5. The most enriched biological processes at 24 h p.i. included endosomal, intracellular and nitrogen compound transports with regulation of vesicles-mediated transport. The GO analysis also showed Golgi organelle and cytoskeleton organizations and interestingly immune response mediated by cells involved in somatic diversification process (immunological memory). Complete GO analysis of biological process, cellular component, and molecular function (BP, CC and MF) for all three time points is provided as supplementary files 4, 5 and 6 (6 h p.i.), files 7, 8 and 9 (15 h p. i.), and files 10, 11 and 12 (24 h p.i.).

**TABLE 5:** Gene Ontology (GO), biological processes (BP) analysis for the top gene targets of piRNAs (time point, RSV 24 h p.i) with *p* values <0.01 or <0.05

Biological process (BP)	Fold enrichment
Golgi organization (GO:0007030)	3.29
activation of GTPase activity (GO:0090630)	2.94
endosomal transport (GO:0016197)	2.11
regulation of small GTPase mediated signal transduction (GO:0051056)	1.93
localization within membrane (GO:0051668)	1.84
protein localization to membrane (GO:0072657)	1.83
regulation of vesicle-mediated transport (GO:0060627)	1.69
intracellular protein transport (GO:0006886)	1.65
cytoskeleton organization (GO:0007010)	1.62
establishment of protein localization (GO:0045184)	1.59
protein transport (GO:0015031)	1.59
protein localization (GO:0008104)	1.56
cellular protein localization (GO:0034613)	1.48
cellular macromolecule localization (GO:0070727)	1.48
macromolecule localization (GO:0033036)	1.48
intracellular transport (GO:0046907)	1.46
establishment of localization in cell (GO:0051649)	1.43
nitrogen compound transport (GO:0071705)	1.43
organelle organization (GO:0006996)	1.42
cellular localization (GO:0051641)	1.39
organic substance transport (GO:0071702)	1.35
cellular component assembly (GO:0022607)	1.29
transport (GO:0006810)	1.28
establishment of localization (GO:0051234)	1.27
cellular component organization (GO:0016043)	1.26
regulation of signaling (GO:0023051)	1.25
cellular component organization or biogenesis (GO:0071840)	1.25
regulation of cell communication (GO:0010646)	1.24
localization (GO:0051179)	1.24
regulation of biological quality (GO:0065008)	1.24
cellular process (GO:0009987)	1.07
biological process (GO:0008150)	1.05
Unclassified (UNCLASSIFIED)	0.71
immune response (GO:0006955)	0.67
G protein-coupled receptor signaling pathway (GO:0007186)	0.5
adaptive immune response (GO:0002250)	0.4

### Clustering of piRNA expression profiles

**FIGURE 4:**
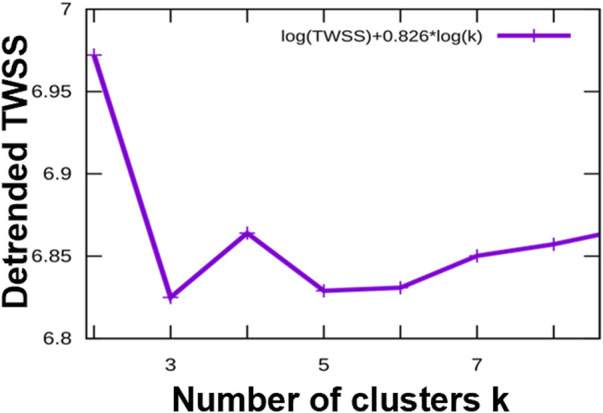
The elbow test indicates that the most informative representations of the categories of temporal profiles of the differentially regulated piRNAs are classifications into three clusters or into five clusters. *X*-axis: number of clusters, *Y*-axis: detrended (residual) *Total Within Sum of Squares*, a measure of structure unexplained by the clustering.

**FIGURE 5:**
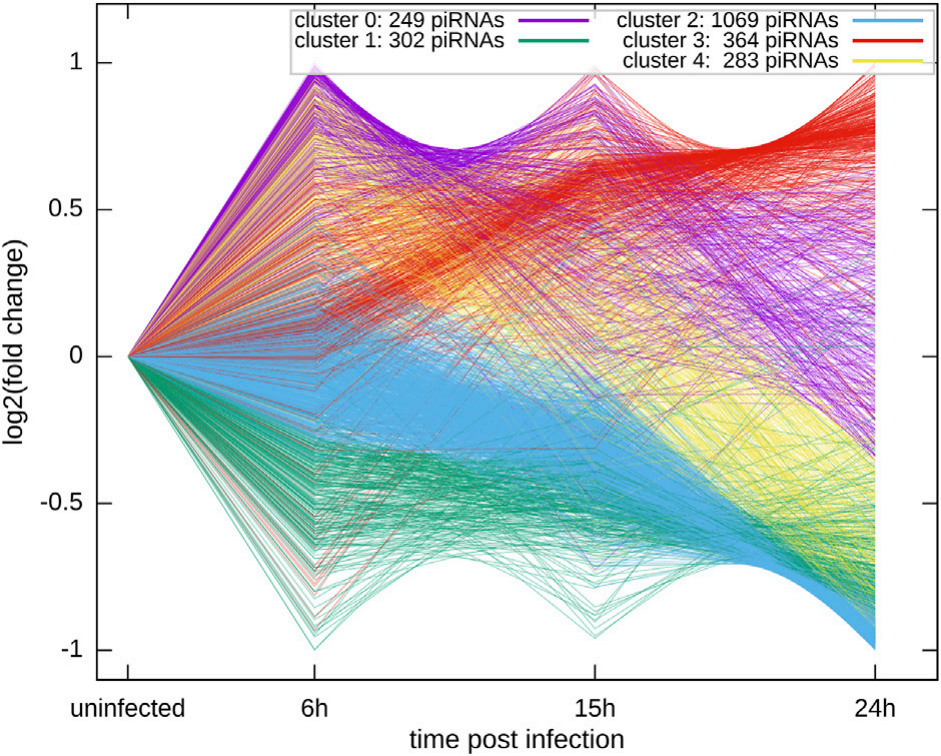
The k-means clustering of the temporal profiles of *X*-axis: time post-infection with RSV (cluster zero represents uninfected cells). *Y*-axis: normalized expression profile, averaged over the replicates, and shown in the log_2_ scale.

To identify common patterns of time-dependent responses to infection, we clustered the significantly regulated expression profiles. Specifically, we used the piRNAs with a significant change at *p* < 0.01 in at least one of the time points, p.i., compared to control. The expression profiles were converted to the log2 scale, averaged over the six replicates, and normalized to levels in uninfected cells and root mean square deviation (rmsd) was equal to 1, rmsd = 1. The preprocessed data were clustered using an in-house implementation of the Hartigan-Wong k-Means algorithm. After conducting an elbow test as shown in Figure 4, we assessed that the most informative clustering was either into three or five groups of temporal profiles. The results of the clustering into five groups are shown in Figure 5. The five basic types of behavior are early (6 h) upregulation, followed by remaining in the upregulated, or uninfected-base level (cluster 0), early downregulation, remaining downregulated (cluster 1), steady downregulation (cluster 2), steady upregulation (cluster 3), and early upregulation, followed by strong downregulation (cluster 4). The complete list of piRNAs for both type of clustering, three or five groups, is included in the supplementary files 13 and 14 named “clustered_list_3. xlsx, and clustered_list_5. xlsx”.

Each of the clusters of temporal profiles have a distinct set of potential targets of the piRNAs in the cluster. Moreover, the targets are associated with enrichment of different functional categories that can be expressed in terms of Gene Ontology biological process enrichments. For example, the early upregulated cluster 0 is enriched with targets with a role in protein transport and localization. The targets of early downregulated piRNAs (cluster 1) are enriched in organelle organization, including Golgi, cytoplasm, and membranes, but depleted in genes coding for regulatory proteins. The piRNAs steadily upregulated (Cluster 3) have potential targets enriched in regulatory genes and inter-cellular signaling. The full lists of the significantly enriched/depleted GO biological process categories for the five clusters are included in the supplementary file 15. We analyzed if specific clusters shown in Figure 5 correlate with subsets of GO categories. The overlaps, for GO BP categories significant at *p* value <0.01 are listed in Table 6. We found that the early (cluster 0) and the steadily upregulated (cluster 3) piRNAs, for example, were linked to GO subcategories regulating the mediated signal transduction of small GTPase, typically involved in the viral immune response. Many cellular functions such as protein transport, intracellular or membrane protein localization, etc. GO subsets were highly associated with targets of piRNAs from the steady downregulated level (cluster 2), which was also associated with adaptive immune response subset. The piRNA targets from the steadily downregulated or upregulated clusters (2 and three respectively) were interestingly connected to GO subcategories involved in the regulation of cellular signaling or biological quality.

**TABLE 6:** Correlation of Gene Ontology (GO) biological processes (BP) subset categories with the clustering of the temporal profiles of *X*-axis.

**Overlaps of GO BP categories at 6 h RSV p.i**		
GO:0007030	clusters: 1 2 4	Golgi organization
GO:0010256	clusters: 1 2 4	endomembrane system organization
GO:0006996	clusters: 1 2	organelle organization
**Overlaps of GO BP categories at 15 h RSV p.i**		
GO:0007030	clusters: 1 2 4	Golgi organization
GO:0007186	clusters: 1 2	G protein-coupled receptor signaling pathway
**Overlaps of GO BP categories at 24 h RSV p.i**		
GO:0007030	clusters: 1 2 4	Golgi organization
GO:0090630	clusters: 2 4	activation of GTPase activity
GO:0016197	cluster: 2	endosomal transport
GO:0051056	clusters: 0 3	regulation of small GTPase mediated signal transduction
GO:0051668	clusters: 0 2	localization within membrane
GO:0072657	clusters: 0 2	protein localization to membrane
GO:0060627	clusters: 0 2	regulation of vesicle-mediated transport
GO:0006886	clusters: 0 2	intracellular protein transport
GO:0007010	clusters: 2	cytoskeleton organization
GO:0045184	clusters: 0 2	establishment of protein localization
GO:0015031	clusters: 0 2	protein transport
GO:0008104	clusters: 0 2	protein localization
GO:0034613	clusters: 0 2	cellular protein localization
GO:0070727	clusters: 0 2	cellular macromolecule localization
GO:0033036	clusters: 0 2	macromolecule localization
GO:0046907	clusters: 0 2	intracellular transport
GO:0051649	clusters: 0 2	establishment of localization in cell
GO:0071705	clusters: 0 2	nitrogen compound transport
GO:0006996	clusters: 1 2	organelle organization
GO:0051641	clusters: 0 2	cellular localization
GO:0071702	clusters: 0 2	organic substance transport
GO:0022607	clusters: 0 2	cellular component assembly
GO:0006810	cluster: 2	transport
GO:0051234	cluster: 2	establishment of localization
GO:0016043	cluster: 2	cellular component organization
GO:0023051	clusters: 2 3	regulation of signaling
GO:0071840	cluster: 2	cellular component organization or biogenesis
GO:0010646	clusters: 2 3	regulation of cell communication
GO:0051179	cluster: 2	localization
GO:0065008	clusters: 2 3	regulation of biological quality
GO:0009987	cluster: 2	cellular process
GO:0008150	cluster: 2	BP
GO:0006955	clusters: 2 4	immune response
GO:0007186	clusters: 1 2	G protein-coupled receptor signaling pathway
GO:0002250	cluster: 2	adaptive immune response
GO:0050906	cluster: 2	detection of stimulus involved in sensory perception
GO:0009593	clusters: 1 2	detection of chemical stimulus
GO:0007606	cluster: 2	sensory perception of chemical stimulus
GO:0007608	cluster: 2	sensory perception of smell
GO:0050907	cluster: 2	detection of chemical stimulus involved in sensory perception
GO:0050911	cluster: 2	detection of chemical stimulus involved in sensory perception of smell

## Discussion

The present study is the first to report changes in piRNAs expression in response to RSV infection of human primary airway epithelial cells. piRNAs are the largest class of sncRNA molecules expressed in animal cells and they can be derived from multiple sources, including transposons, mRNAs, and ncRNAs, such as lncRNAs, snoRNAs and tRFs. Among these piRNAs, only the function of transposon-derived piRNAs in gonadal cells has been relatively well studied (Weick and Miska, 2014). piRNAs have been shown to play a crucial role to safeguard genome, maintaining the genome complexity and integrity, as they suppress the insertional mutations caused by transposable elements, however, there is growing evidence of their role in controlling gene expression in somatic cells as well. The biogenesis and function of piRNAs is associated with three different proteins, namely PIWI, Argonaute-3 (AGO3), and Aubergine (AUB). PIWI proteins are found both in somatic cells and germ cells, while AGO3 and AUB are observed primarily in germ cells. Precursors of piRNAs are exported from the nucleus into the cytoplasm to be further processed into smaller sequences and reach their partners to form piRNA-PIWI complexes. These complexes can migrate back to the nucleus to block the transcription of target genes. There are several mechanisms underlying piRNA-mediated gene regulation. The better characterized one relates to epigenetic changes, including DNA methylation and histone modifications, whereas piRNA–PIWI complex can bind to the promoter region of a gene and favors recruitment of methylation factors, enabling transcriptional silencing. Some examples of this mode of gene regulation in somatic cells are the silencing of killer immunoglobulin-like receptor, the immune receptor CD1A and the antioxidant enzyme glutathione-S-transferase (Zhang et al., 2016; Zhang et al., 2020). In addition, piRNAs can also regulate gene expression at the post-transcriptional level, as in the case of piRNA-30840 which binds to IL-4 pre-mRNA, leading to its degradation (Zhong et al., 2015), and piR-FTH1, a piRNA found in breast cancer cells, which downregulates ferritin heavy chain mRNA (Balaratnam et al., 2018). One last mechanism by which piRNAs regulate gene expression has been recently identified and it related to the interaction with the cellular RNA methylation machinery, leading to changes in mRNA m6A methylation, a post-transcriptional modification of mRNAs in mammals that plays a fundamental role in RNA stability and translation (Gao et al., 2020).

Although the role of piRNAs was initially confined to gonad development, recent studies have identified specific expression profile of piRNAs from multiple organs and cell types. Currently, piRNA-disease association data are available for several diseases, including various types of cancers, cardiovascular diseases, autoimmune and neurodegenerative disorders (Muhammad et al., 2019). Surprisingly, almost nothing is known regarding piRNA generation/function and PIWI proteins in the context of viral infections. In our recent published study, analysis of RNA content of exosomes isolated from RSV-infected airway epithelial cells identified piRNAs as an important component of the exosomal cargo (Chahar et al., 2018) Next-generation sequencing analysis revealed that 52 piRNAs were commonly present in both mock and RSV exosomes, with 28 upregulated and 24 downregulated piRNAs in RSV exosomes, with 3 and 19 piRNAs that uniquely present in mock and RSV exosomes, suggesting a biological function for this class of sncRNAs during infection. In the current study, we show for the first time that RSV infection can lead to significant changes in piRNAs in infected SAE cells in a time-dependent manner. Our piRNA microarray analysis revealed an enrichment in numbers of up- and down-regulated piRNAs in airway epithelial cells upon RSV infection compared to mock cells, with the highest numbers of differentially expressed piRNAs detected at 24 h p. i. With RSV. Significant changes in the expression profiles of piRNAs was recently reported for Coxsackievirus B3 (CVB3) infection in human HeLa cells using high-throughput sequencing. Similar to RSV, CVB3 infection was associated with time-dependent changes in piRNAs numbers, although the highest number of differentially expressed piRNAs was observed at the early time points post-infection (Yao et al., 2020). In an animal model of ducks infected with avian influenza virus, piRNAs represented the highest class of sncRNAs expressed after infection compared to other snRNA classes, such as microRNAs (miRNAs) and small nucleolar RNAs (snoRNAs) (Samir et al., 2020). There were major organ-specific differences in sncRNA populations at baseline and substantial reprogramming of all sncRNA classes throughout infection.

Target prediction analysis suggested that piR-38587 could possibly target AP5Z1, the adaptin proteins 5 (AP5). Very little is known on AP5 because it is the most recently identified adaptin complex. Although AP5 has not been investigated in the context of viral infection, AP2, another type of AP complex has been shown to function as a target of ocular infection upon human adenovirus species D type 37 (HAdV-D37) infection (Lee et al., 2020) or be involved in the entry process of human enterovirus 71 (HEV71) (Hussain et al., 2011). Similarly, piR-4095 target includes DDX4 which is an RNA helicases enzyme. Different DDX enzymes, as DDX1, DDX3X, and DDX5, have been known to play important roles in the replication cycle of coronaviruses (Taschuk and Cherry, 2020). piR-57640 could target STAT2 which is involved in RSV infection. It is known that RSV is a potent inducer of type I interferon release from infected airway epithelial cells (Garofalo et al., 1996), and the virus specifically downregulates human STAT2 protein expression, thus enabling RSV to evade the host type I interferon response (Elliott et al., 2007). The Gene Ontology functional classification found that the most significant groups of targets of regulated piRNAs are related to long noncoding RNA, DNA- and protein-binding, transcriptional and post-transcriptional regulation.

Although piRNA function in response to an infection in mammalian cells has not been explored yet, murine neural stem cells (NSCs) were recently shown to release piRNA-containing exosomes/microvesicles with antiviral immunity (Ikhlas et al., 2022). When NSCs were exposed to RNA fragments of SARSCoV-2 genome, the derived extracellular vesicles displayed enhanced antiviral activity. The increased antiviral effect of these vesicles was associated with increased expression of piRNA species some of which were predicted to target SARS-CoV-2 genome. Knockout of piRNA-interacting protein PIWIL2 in NSCs led to a reduction in the induced antiviral effect, suggesting that the PIWI-piRNA system is important for these antiviral actions (Ikhlas et al., 2022). A recent study of bacterial pneumonia using a knockout mouse for MIWI2, the mouse homologue of human PIWIL4, revealed an important role of this piwi protein in lung innate immune response. MIWI2 was expressed by a subpopulation of multiciliated airway epithelial cells, and its expression was increased following infection with *S*. *pneumoniae* (Wasserman et al., 2017). RNA sequencing found that MIWI2-positive cells expressed a significantly different transcriptome compared with neighboring cells that did not express MIWI2, and MIWI2 *−/−* was associated with enhanced innate immune responses and faster bacterial clearance (Wasserman et al., 2017).

In conclusion, our study provides insights into changes of the expression profile of piRNAs during RSV infection and highlight the need for more detailed investigations of the piRNA/PIWI protein pathway in the context of viral infection to establish their potential role in regulation of cellular responses. A better understanding of this pathway could potentially identify novel therapeutic targets for future piRNA-mediated strategies to modulate RSV-associated lung disease, as well as potential biomarkers for disease diagnosis and prognosis.

## Data Availability

The original contributions presented in the study are included in the article/Supplementary Material, further inquiries can be directed to the corresponding author.
