# Supplementary figures and images for: Respiratory syncytial virus infection changes the piwi-interacting RNA content of airway epithelial cells
Source: Front Mol Biosci. 2022 Sep 8;9:931354. doi: 10.3389/fmolb.2022.931354 (PMC9493205; doi:10.3389/fmolb.2022.931354)

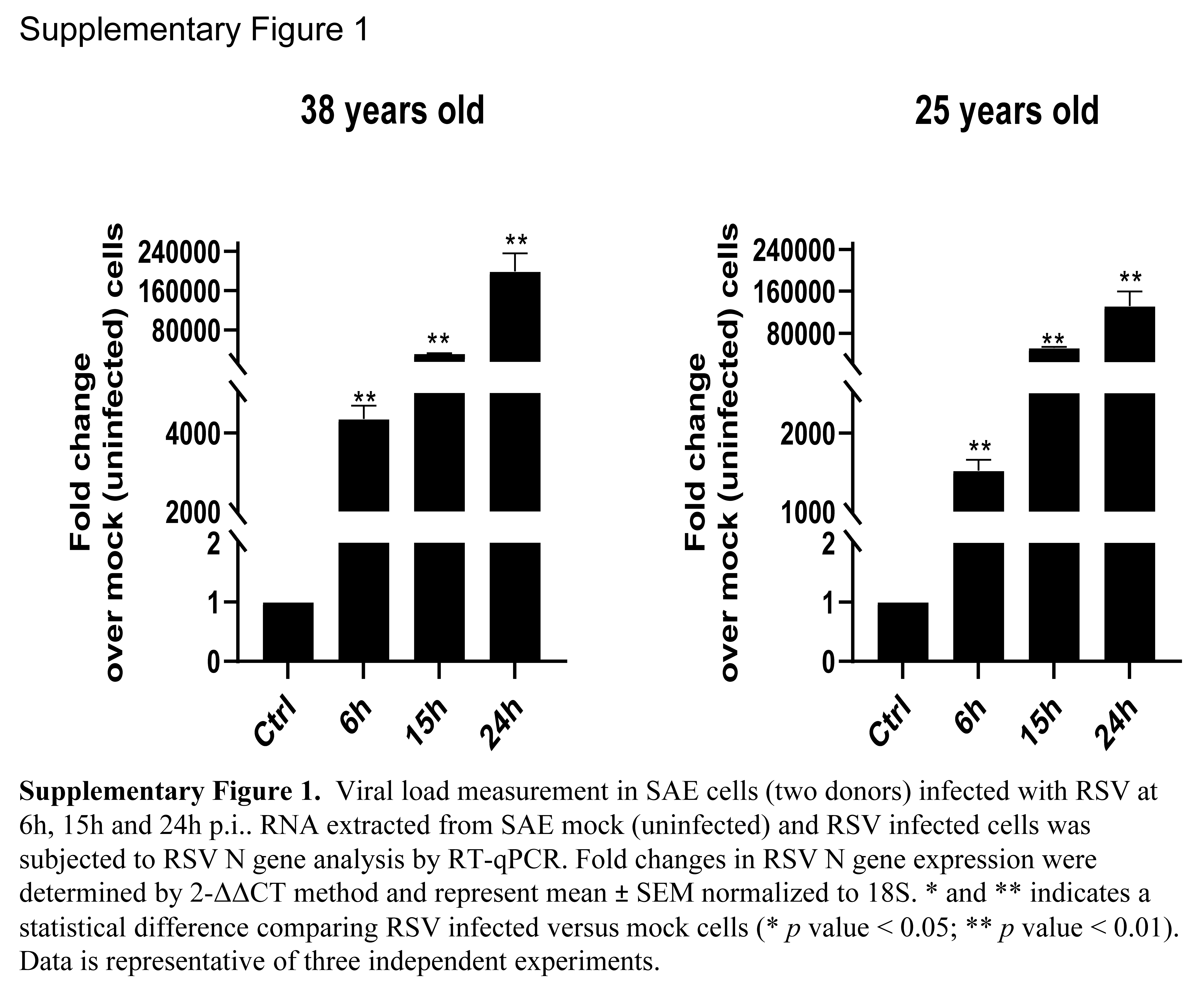

Supplement: Supplementary file 1 [file Image1.TIF]
